# Development of a D genome specific marker resource for diploid and hexaploid wheat

**DOI:** 10.1186/s12864-015-1852-2

**Published:** 2015-08-28

**Authors:** Yi Wang, Thomas Drader, Vijay K. Tiwari, Lingli Dong, Ajay Kumar, Naxin Huo, Farhad Ghavami, M. Javed Iqbal, Gerard R. Lazo, Jeff Leonard, Bikram S. Gill, Shahryar F. Kianian, Ming-Cheng Luo, Yong Q. Gu

**Affiliations:** Western Regional Research Center, USDA-ARS, Albany, CA 94710 USA; Department of Plant Sciences, University of California, Davis, CA 95616 USA; Department of Crop and Soil Science, Oregon State University, Corvallis, OR 97331 USA; Wheat Genetic Resource Center, Department of Plant Pathology, Kansas State University, Manhattan, KS 66506 USA; Department of Plant Sciences, North Dakota State University, Fargo, ND 58108 USA; Molecular Breeding and Genomics Technology Laboratory, BioDiagnostics Inc., River Falls, WI 54022 USA; Cereal Disease Laboratory, USDA-ARS, Minneapolis, MN 55108 USA

**Keywords:** Wheat deletion bins, Molecular markers, Repeat junction markers, NimbleGen array, Recombination, Genetic map

## Abstract

**Background:**

Mapping and map-based cloning of genes that control agriculturally and economically important traits remain great challenges for plants with complex highly repetitive genomes such as those within the grass tribe, *Triticeae*. Mapping limitations in the *Triticeae* are primarily due to low frequencies of polymorphic gene markers and poor genetic recombination in certain genetic regions. Although the abundance of repetitive sequence may pose common problems in genome analysis and sequence assembly of large and complex genomes, they provide repeat junction markers with random and unbiased distribution throughout chromosomes. Hence, development of a high-throughput mapping technology that combine both gene-based and repeat junction-based markers is needed to generate maps that have better coverage of the entire genome.

**Results:**

In this study, the available genomics resource of the diploid *Aegilop tauschii*, the D genome donor of bread wheat, were used to develop genome specific markers that can be applied for mapping in modern hexaploid wheat. A NimbleGen array containing both gene-based and repeat junction probe sequences derived from *Ae. tauschii* was developed and used to map the Chinese Spring nullisomic-tetrasomic lines and deletion bin lines of the D genome chromosomes. Based on these mapping data, we have now anchored 5,171 repeat junction probes and 10,892 gene probes, corresponding to 5,070 gene markers, to the delineated deletion bins of the D genome. The order of the gene-based markers within the deletion bins of the Chinese Spring can be inferred based on their positions on the *Ae. tauschii* genetic map. Analysis of the probe sequences against the Chinese Spring chromosome sequence assembly database facilitated mapping of the NimbleGen probes to the sequence contigs and allowed assignment or ordering of these sequence contigs within the deletion bins. The accumulated length of anchored sequence contigs is about 155 Mb, representing ~ 3.2 % of the D genome. A specific database was developed to allow user to search or BLAST against the probe sequence information and to directly download PCR primers for mapping specific genetic loci.

**Conclusions:**

In bread wheat, aneuploid stocks have been extensively used to assign markers linked with genes/traits to chromosomes, chromosome arms, and their specific bins. Through this study, we added thousands of markers to the existing wheat chromosome bin map, representing a significant step forward in providing a resource to navigate the wheat genome. The database website (http://probes.pw.usda.gov/ATRJM/) provides easy access and efficient utilization of the data. The resources developed herein can aid map-based cloning of traits of interest and the sequencing of the D genome of hexaploid wheat.

**Electronic supplementary material:**

The online version of this article (doi:10.1186/s12864-015-1852-2) contains supplementary material, which is available to authorized users.

## Background

Complex genomes such as that of wheat are a major hurdle in identification of genes controlling agriculturally important traits for crop improvement. Hexaploid wheat (*Triticum aestivum* L., 2n = 6x = 42, AABBDD) arose from two hybridization events [1]. The wild diploid wheat (*T.urartu*) AA genome progenitor, hybridized with the BB genome ancestor (unknown but most closely related to the goat grass, *Aegilops speltoides*) to form the wild emmer wheat (*T. dicoccoides*, 2n = 28; AABB). *T. dicoccoides* was subsequently hybridized with another goat grass *Ae. tauschii,* DD genome, to form spelt wheat (*T. aestivum ssp. spelta*). After selection and spontaneous mutations the emmer and spelt species evolved into the current tetraploid durum wheat (*T. aestivum ssp. durum*, genomes AABB), and hexaploid bread wheat (*T. aestivum*, genomes AABBDD). These two wheat species combined are ranked in the top five grains of the global food supply, estimated at 37 % of total human consumption [2].

The great importance of wheat as a food crop has led to genomics research in order to develop useful tools for breeding and genetic improvement studies. Numerous tools have been developed to aid in the identification of genes encoding agriculturally desirable traits. Recent advances in sequencing technologies have produced cost effective methods to generate high sequence coverage of large genomes [3, 4]. Ongoing efforts towards sequencing the hexaploid wheat genome has already generated useful sequence resources, including whole genome shotgun sequences and individual chromosome sequences of a wheat reference cultivar, Chinese Spring [5, 6]. To complete the sequence assembly of the wheat genome, one of the big challenges is to order and orientate resulting contiguous sequences onto respective chromosomes via high-resolution integrated genetic and physical maps to generate a high-quality reference genome sequence.

Gene-based markers are often more useful for mapping and provide information related to gene structure and organization within the genomic regions of interest. However, the use of gene-based markers might provide a limited framework for construction of genome-wide maps, particularly in large and complex genomes. In hexaploid wheat, genes are not evenly distributed along the chromosomes with large stretches of uninterrupted non-coding spaces. Genes from three homeologous chromosomes are often conserved, and sometimes, it is difficult to assign the three homeologous copies to individual chromosomes. Moreover, 30 % of the genes are in recombination poor regions [7]. Taken together, gene-based markers might not be able to produce a complete framework in polyploid wheat for anchoring and ordering sequence contigs along the chromosomes.

The prevalence, structure, and insertion patterns of transposable elements in the wheat genome provide useful resources for developing unique marker system that has been shown to have the potential in genetics, genomics, and marker-assisted selection [8–11]. Since the insertion sites of transposable elements are unique and often show high polymorphism even among wheat varieties, markers designed based on the repeat junction sites have also been termed "insertion site-based polymorphism (ISBP) markers. Previous studies indicated that these markers not only showed high insertion polymorphism, but also can be efficiently converted into SNP markers for high-throughput genetic or diversity mapping due to much higher nucleotide polymorphism in the junction sites as compared to the gene regions [11]. Therefore, this type of markers is a critical resource that can be used to saturate genetic maps, genotype elite cultivars, and develop tightly linked markers to traits for marker assisted selection [11]. The relationship between a transposable element and its surrounding insertion site is unique within a genome or genome specific and can behave as a low copy locus. Repeat junction sites are considered to be ubiquitous, with less biased distribution along the chromosomes [9]. The unique repeat junction site loci can be physically mapped to specific chromosomes and within chromosome bins using deletion lines. Previously, we have demonstrated that 90 % of PCR-based repeat junction markers derived from *Ae. tauschii* can be specifically mapped to the respective D chromosomes [8, 9] in the hexaploid wheat, with no need for further genomic assignment, demonstrating its potential in development of genome-wide molecular markers for mapping and genetic diversity studies in large and complex genomes [10–12]. Therefore, development of a high-throughput mapping technology to map both gene-based and repeat junction-based markers is needed to generate maps that have better coverage of the entire genome.

NimbleGen arrays for comparative genomic hybridizations (CGH) have been used in maize, Arabidopsis, soybean, rice, and barley [13–17]. Traditional use of these arrays involves the tiling of overlapping probes across regions of the genome, and the subsequent detection of copy number variants (CNV), and presence/absence variations (PAV) [18, 19]. The tiled probes are most often in gene rich regions or specifically within genes themselves. As previously mentioned, gene based detection limits the number of physical markers and does not encompass the entire genome. By use of both gene based and repeat junction markers, this limitation could be overcome. Recently, CGH array was developed to map wheat 7B sequence markers including repeat junction sequences into deletion bins to construct high density deletion bin maps [20]. In this study, we designed a NimbleGen array containing both repeat junction sequences and gene-based markers from the *Ae. tauschii* sequences and mapped them to the D genome of hexaploid wheat. The development of the NimbleGen array allowed the construction of a physical bin map from the known deletion bin lines of the D genome of hexaploid wheat. The mapping of a large number of both gene-based markers and repeat junction sites to delineated bins in the D genome provides a useful resource that could greatly facilitate mapping genes/QTL traits of interest in wheat.

## Results

### Identification and screening of repeat junction probes

**Fig. 1:**
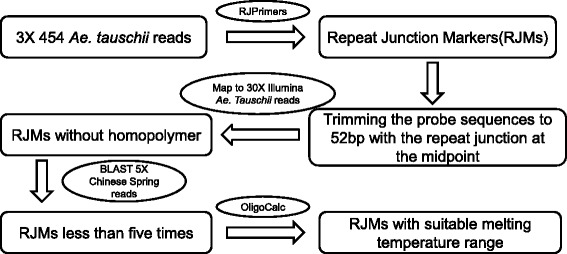
Schematic presentation of a pipeline for the development of RJMs from *Ae. tauschii* sequences

Figure 1 depicts a schematic representation of a pipeline used for developing a NimbleGen array for the hexaploid wheat mapping. A total of ~ 9,000,000 Roche 454 reads representing ~ 1x coverage of the *Ae. tauschii* genome (~4.2 G) were used for the repeat junction analysis. After running the reads on RJPrimers software [8], a total of 987,000 repeat junctions were identified. To improve the quality of the probes on the NimbleGen array for mapping, we then implemented a four step process for probe selection.

1. Probes for the NimbleGen array were designed to have a fixed length of 52 bp with the repeat junction at the midpoint. Our pilot experiment with various probe lengths of repeat junction sequences on NimbleGen array suggested that probes with a 52-bp length provides the best performance in the mapping data (data not shown).
2. The probe sequence accuracy was validated by mapping the sequences to the *Ae. tauschii* Illumina reads representing 30X genome coverage. This step also eliminated the potential homopolymer problems and other sequence errors in the Roche 454 reads. In addition, through use of this step, we removed probes containing homopolymer regions (*n* > 3),
3. Probes with high copy number in the genome were removed by BLASTN of the 52 bp probe sequences against the 5X Chinese Spring genome shotgun reads generated by Roche 454 [21]. If a probe sequence was exactly matched more than five times, this probe was considered to have more than one copy and removed. Since the Chinese Spring reads were used in the process, this step also eliminated those repeat junction probes that were not shared between the *Ae. tauschii* and Chinese Spring D genomes, providing a probe set that can be used in mapping of both *Ae. tauschii* and the hexaploid wheat. Through this sequence mapping analysis, 89 % of the 52 bp probe sequences from *Ae. tauschii* were found to be single copy and had perfect matches to Chinese Spring. This agreed well with our previous mapping result that showed 90 % of PCR-based RJMs derived from *Ae. tauschii* can be mapped to the D genome chromosomes of Chinese Spring [8].
4. We also used the OligoCalc [22] program to determine the melting temperature for each markers. Marker sequences with a Tm value between 76 °C to 83 °C were maintained. This step ensured all the probes would have similar hybridization dynamics.

In order to identify probes that would perform well for mapping to the D genome, we conducted a pilot experiment by screening probes on a 3*720 K CGH array using genomic DNAs from the hexaploid Chinese Spring wheat (AABBDD) and from the tetraploid durum wheat (AABB) missing the D genome. Probes that showed at least 20 % signal reduction in durum sample as compared to the Chinese Spring in replicated hybridization experiments were considered as reliable and D genome specific probes. After these processes, we finally selected 31,205 RJMs that were used as probes to be fabricated in a 12*135 K CGH array for mapping.

### Types and distribution of repeat junction probes

**Fig. 2:**
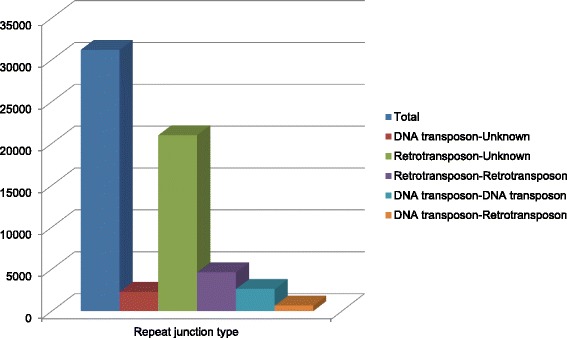
Distribution of different types of repeat junctions of the *Ae. tauschii* RJMs on the NimbleGen array*.* The probe sequences representing repeat junction markers on the NimbleGen array were analyzed using the RJPrimer program to determine the types of the repeat junctions. The number in the vertical axis indicates the occurrence of each repeat junction type

Repeat junction markers can be placed into different categories depending on two repeat sequences involved in the junction region [8]. Analysis of different types of repeat junctions could allow for better understanding of activities of repeat sequence elements in the genome. We further analyzed the repetitive sequence composition in these 31,205 repeat junction site sequences on the array using the RJPrimer pipeline [8]. A majority of the repeat junctions were grouped into the ‘retrotransposon-unknown’ category (21,005) (Fig. 2). The 'unknown' category used here implied that one of the two sequences in the junction region did not have a significant match to the repeat sequence database used in the RJPrimer pipeline (e value cutoff less than e-10). Therefore, if a retrotransposon element was inserted into a genic or low copy sequence region, it would be grouped into this ‘retrotransposon-unknown’ category. However, the high number of this category could also be due to the fact that the current collection of repeat elements in the database only represented a marginal portion of the elements for the wheat genome. We also noticed that the lowest number of repeat junction types were the 'DNA transposon-retrotransposon' category. Given the high percentage of retroelements in the genome [3], we expected the number of this category to be higher than the DNA transposon-DNA transposon category. However, it is not clear if such a result is due to the tendency for a transposable element to insert itself into a similar class of repeat element during the transposition process (in this case, a DNA transposon inserted into another DNA transposon).

### Gene-based marker probes

Recently, a genetic map containing 7,185 SNP-based markers was constructed for the *Ae. tauschii* genome [21]. For this map, a majority of the SNP markers were derived from gene sequences. To include these gene-based marker sequences in the NimbeGen array, we anchored the SNP-based marker sequences to the *Ae. tauschii* shotgun genome assembly to identify the gene sequences containing these SNP markers [3]. These gene sequences were then extracted and used to design gene-based marker probes for the NimbleGen array. We included 6,348 gene regions in total for the NimbleGen array with 1 to 3 probes represented for each gene region. Therefore, the final NimbleGen array was constructed consisting of 15,016 gene marker probes for 6,348 genes and 31,205 repeat junction probes with each probe replicated three times on the array.

### Marker assignment to chromosomes with NimbleGen array

To examine the mapping accuracy and marker distribution among the D chromosomes, we first mapped probe sequences on the NimbleGen array onto specific wheat D chromosomes by using seven Chinese Spring (CS) nulli-tetrasomic lines. These lines represent missing each pair of the D chromosomes that were replaced by an extra pair of their respective homoeologous chromosome. These genetic stocks have been widely used to assign marker/genes to specific wheat chromosomes [23]. Assuming that a marker/probe is specific to a locus on a specific chromosome, it will display loss of signal with its corresponding substitution line, while the control and all other substitution lines will show a normal signal ratio. In our study, each line was hybridized in duplicate and analyzed for marker signal loss (20 % signal loss, *p* < 0.05). Signal loss was initially evaluated at 50 % and the stringency was decreased in increments of 10 % until reaching a 20 % loss of signal.

Nulli-tetrasomic analysis resulted in mapping of 41,610 sequence probes (12,417 from gene markers and 29,193 from repeat junction sites) on seven D genome chromosomes, which was ~ 90 % of the total probes used for array hybridization. The total number of markers/probes (gene and repeat junction sites) mapped on individual D chromosomes were found to be 5,482 (1D), 6,069 (2D), 6,606 (3D), 5,778 (4D), 6,553 (5D), 4,849 (6D), and 6,273 (7D) (Table 1). Out of 15,016 gene-based probes which represented 6,348 genes, 12,417 (5,962 genes) were mapped on nullisomic-tetrasomic lines for the D genome chromosomes. Similarly, out of total 31,205 repeat junction probes, 29,193 were mapped on nullisomic-tetrasomic lines of the D genome. There were ~2000 probes which could not be assigned to any of the D genome chromosomes. Individual chromosome-wise distributions of gene-based and repeat junction probes are presented in Table 1. A total of 35,118 (5,962 genes presented by multiple probes from each genes and 29,193 repeat junction sites) probes were mapped on the D chromosomes in this study. If the estimated genome size of ~4.9 Gb is used to represent the D genome contribution for both diploid and hexaploid wheat, ~35,000 sequence probes generated in this study roughly provide one marker at every ~150 Kb (~7 markers/Mb) interval (Table 1).

**Table 1:** Distribution of gene and repeat junction markers on the seven D genome chromosomes in Chinese Spring

Chromosome	Size (Mb)	RJMs mapped on nullisomic lines	Gene -based probes on nullisomic lines	Unique genes covered by Gene-based probes	Total no. of RJM+ Gene-based probes
1D	604	3921	1561	743	4664
2D	727	4146	1923	923	5069
3D	770	4453	2153	1031	5484
4D	648	4366	1412	672	5038
5D	748	4491	2062	982	5473
6D	712	3551	1298	618	4169
7D	727	4265	2008	956	5221
Total	4936	29193	12417	5925	35118

### Mapping markers to delineated bins of the D genome

**Fig. 3:**
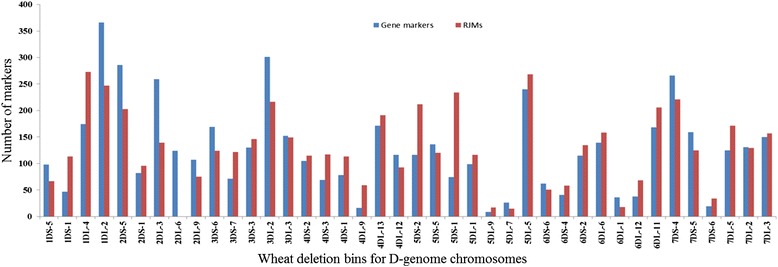
Distribution of unique gene-based and repeat junction markers mapped in each wheat deletion bin lines of the D genome chromosomes**.** The NimbleGene array was used to anchor markers to delineated bins as indicated. The vertical axis indicates the number of markers in each bin. Gene-based and repeat junction markers in each bin are represented by different colour bars

To increase the utility of this mapping resource for wheat research, we mapped sequence probes to specific chromosome regions by hybridizing the array using 40 deletion bin lines of the D genome chromosomes. As shown in Fig. 3, out of 12,417 gene based probes (mapped on nullisomic-tetrasomic lines), 10,892 probes (87 %) were mapped on 40 deletion bins of the D genome. The description of the deletion lines and their fragment lengths are presented in Table 2. The remaining 1,525 (13 %) (892 unique gene markers) probes were mapped on nullisomic-tetrasomic lines of the D genome, but failed to map on deletion bin lines. For the repeat junction probes, only 5,171 (18 %) could be confidently mapped to the deletion bins. In total, we mapped 16,063 probes (10,892 gene based probes + 5,171 repeat junction sites) on 40 deletion bins of the D genome chromosomes (Fig. 3). Gene based probes (10,892) corresponded to 5,070 gene markers. Along with the 5,171 repeat junction probes, we mapped 10,241 unique loci on deletion bins of the wheat D genome. On average, 256 (with a range of 26–613 markers) unique markers (gene + repeat junction sites) were mapped per deletion bin with lowest and highest numbers in deletion bins, 5DL-9 and 1DL-2, respectively (Fig. 3). Since the fragment length of the deletion bins used in this study can be estimated by the method described in Tiwari et al. [24] and the estimated size of the *Ae. tauschii* genome ranges from 4.02 to 4.95 Gb [25, 26], we roughly estimated the total fraction of the D genome analyzed. In total, the deletion bin lines used encompassed ~2.5Gb for the D chromosomes (Table 2), which is ~50 to 62 % of the entire D genome. A total of 10,241 mapped loci (gene markers and repeat junction sites) provide a marker at every ~250 Kb of the genome assayed in this study (Table 2).

**Table 2:** Wheat deletion bins, their estimated sizes and number of markers assigned to the bins in the D- genome chromosomes

Chromosome	Deletion bins	Physical location of deletion bins	Fragment length analyzed (Mb)*	Gene based markers mapped	RJMs mapped
	1DS5-0.70-1.00	Terminal bin	67.2	98	**67**
	1DS1-0.59-0.70	Interstitial bin	24.64	47	**113**
**1D**	***1DS3-0.48-0.59 - C-1DL4-0.18***	***Pericentromeric and centromeric bins*** ^***$***^	***200.74***	***58***	***3221***
	1DL4-0.18-0.41	Interstitial bin	87.63	174	**273**
	1DL2-0.41-1.00	Terminal bin	224.79	366	**247**
	2DS5-0.47-1.00	Terminal bin	167.48	82	**96**
	2DS1-0.33-0.47	Interstitial bin	44.24	286	**203**
**2D**	***C-2DS-0.33- C-2DL3-0.49***	***Pericentromeric and centromeric bins***	***305.67***	***65***	***3633***
	2DL3-0.49-0.76	Interstitial bin	110.97	259	**139**
	2DL9-0.76-1.00	Terminal bin	98.64	231	**75**
	3DS6-0.55-1.00	Terminal bin	144.45	240	**246**
	3DS3-0.24-0.55	Interstitial bin	99.51	130	**146**
**3D**	***C-3DS3-0.24 - C-3DL2-0.27***	***Pericentromeric and centromeric bins***	***198.27***	***208***	***3696***
	3DL2-0.27-0.81	Interstitial bin	242.46	301	**216**
	3DL3-0.81-1.00	Terminal bin	85.31	152	**149**
	4DS2-0.81-1.00	Terminal bin	43.89	105	**115**
	4DS3-0.67-0.81	Interstitial bin	32.34	69	**117**
	4DS1-0.53-0.67	Interstitial bin	32.34	78	**113**
**4D**	***C-4DS1-0.53- C-4DL9-0.31***	***Pericentromeric and centromeric bins***	***251.39***	***117***	***3678***
	4DL9-0.31-0.56	Interstitial bin	104	16	**59**
	4DL-13-0.56-0.71	Interstitial bin	62.4	171	**191**
	4DL12-0.71-1.00	Terminal bin	120.64	116	**93**
	5DS2-0.78-1.00	Terminal bin	56.76	116	**212**
	5DS5-0.67-0.78	Interstitial bin	28.38	136	**120**
	5DS1-0.63-0.67	Interstitial bin	10.32	74	**234**
**5D**	***C-5DS1-0.63- C-5DL1-0.60***	***Pericentromeric and centromeric bins***	***456.54***	***282***	***3509***
	5DL1-0.60-0.74	Interstitial bin	68.6	99	**116**
	5DL9-0.74-0.76	Interstitial bin	9.8	9	**17**
	5DL5-0.76-1.00	Terminal bin	117.6	266	**283**
	6DS6-0.99-1.00	Terminal bin	3.24	62	**51**
	6DS4-0.79-0.99	Interstitial bin	64.8	41	**58**
	6DS2-0.45-0.79	Interstitial bin	110.16	115	**135**
**6D**	***C-6DS2-0.45 - C6DL5-0.29***	***Pericentromeric and centromeric bins***	***258.61***	***19***	***2857***
	6DL6-0.29-0.47	Interstitial bin	70.02	139	**158**
	6DL1-0.47-0.68	Interstitial bin	81.69	36	**18**
	6DL12-0.68-0.74	Interstitial bin	23.34	38	**68**
	6DL11-0.74-0.80	Interstitial bin	23.34	168	**206**
	7DS4-0.61-1.00	Terminal bin	148.59	285	**255**
	7DS5-0.36-0.61	Interstitial bin	95.25	159	**125**
**7D**	***C-7DS5-0.36 - C-7DL5-0.30***	***Pericentromeric and centromeric bins***	***240.96***	***106***	***3394***
	7DL5-0.30-0.61	Interstitial bin	107.26	125	**171**
	7DL-2-0.61-0.82	Interstitial bin	72.66	131	**129**
	7DL3-0.82-1.00	Terminal bin	62.28	150	**157**

*The sizes of deletion bins were estimated based on the method used by Tiwari et al. [24]. The fragment length analyzed for individual chromosomes were calculated based on the accumulated length of each deletion bin in the chromosome

^$^The pericentromeric and centromeric deletions bins presented here (bold and italicized) were not used in hybridization experiments. Markers in these bins were placed after subtracting total number of markers mapped on deletion bin lines out of total number of markers mapped on nullisomic-tetrasomic lines for a given chromosome

### Validation of marker assignment to specific chromosomes and bins

To evaluate the mapping accuracy, we analyzed our probes and mapping results using two approaches:

(i) We BLASTN compared the NimbleGen mapped probe sequences against the individual Chinese Spring chromosome sequence data available in the public databases (https://urgi.versailles.inra.fr/download/iwgsc/). We assumed that a marker mapped to a specific chromosome by the NimbleGen array would map to the same chromosome by this BLASTN analysis. The BLAST results showed that 17,453 probes on the array could be mapped to a specific chromosome as defined by a single perfect match of the probe sequence against the database. When the chromosome assignment results generated by the NimbleGen mapping and BLAST analysis were compared,13,154 (82 %) were assigned to the same chromosome by both methods.
(ii) We also compared our NimbleGen deletion bin mapping result with the previous wheat EST deletion bin map [27]. In the wheat deletion bin map, 7,104 expressed sequence tag (EST) unigenes were mapped by Southern hybridization to chromosome bins using a set of wheat aneuploids and deletion stocks. We downloaded the EST loci data (http://wheat.pw.usda.gov/wEST/binmaps/) and identified that 4,058 EST markers were mapped to different bins in the D genome. Further analysis of the ESTs indicated that they represented only 2,962 non redundant sequences. When they were compared with the gene marker sequences on the NimbleGen array, we found 268 EST sequences were the original source for 494 NimbleGen probes. Among these 494 probes, 209 (60 %) have the same chromosome bin assignment with the EST loci (Additional file 1: Supplement T1). Our percentage of the agreement between the NimbleGen and EST deletion bin maps is comparable with a recent report on wheat chromosome 7B, where ~ 30 % of the NimbleGen assigned bin locations didn't agree with the previous EST mapped result [20].

Our sequence BLAST analysis showed that over 50 % of the probe sequences on the array did not find matches against the assembled Chinese Spring sequence contigs in spite of the fact that these sequences were validated by the Chinese Spring reads generated by Roche 454 data (Fig. 1). Analysis of these unmatched sequences indicated that only 25 % belonged to gene markers and the remaining were repeat junction probes. The much higher percentage of unmatched repeat junction sequences could be explained by the notion that most of the repeat sequences were removed and not included in the original sequence assembly [6]. Therefore, repeat sequences are not well represented in the current assembled wheat genome. In this study, we mapped these unmatched repeat junction site sequences to the D chromosomes or specific deletion bins.

### Genetic distance of deletion bins and ordering gene-based markers within deletion bins

**Fig. 4:**
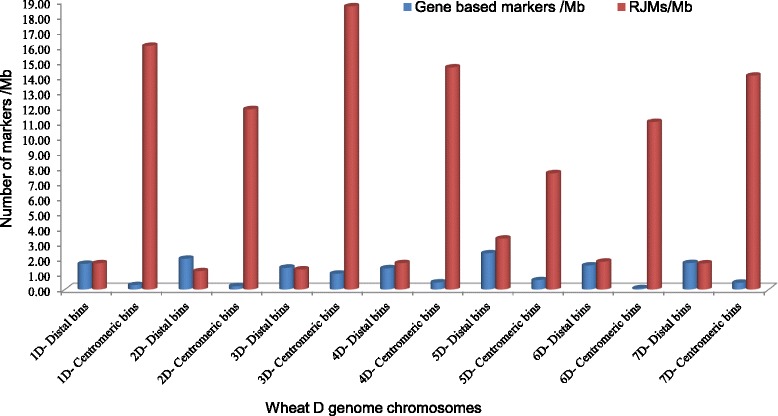
Dot plot of bin-mapped gene markers against their genetic position in the *Ae. tauschii* genetic map. Gene-based markers mapped to individual deletion bins were used to compare with the markers in the *Ae. tauschii* genetic and physical maps [21]. The analysis provided the genetic (x-axis) and physical positions (y-axis) of each marker in each bin along the chromosomes, as well as the order of markers within the bin. The result was used to generate the dot plot. Only the markers in the deletion bins that showed consistent position with the genetic map was included in the dot plot. The gap regions represent the missing bins along the chromosomes

Deletion bins along the chromosome arms have been defined as chromosome segments lying between the breakpoints of two deletion lines. However, the genetic distance of the deletion bins have not been well determined. The recent *Ae. tauschii* genetic map contained 7,185 SNP markers, representing a great resource for the D genome. By comparing genetic and deletion bin maps, we can infer the genetic distance of specific deletion bins on the genetic map (Fig. 4). Clearly, genetic length of each deletion bin is quite different. Generally, deletion bins in the distal regions are better defined due to greater recombination (i.e. have large genetic length) while bins towards the centromeric regions tend to be clustered (i.e. have small genetic length). Therefore, there is no correlation of genetic length with the deletion bin size (p-value < 0.05). The uneven distribution of recombination rate along wheat chromosomes have been widely investigated with recombination increasing gradually from the centromeres to the telomeres [28]. Because of this recombination gradient, it has been estimated that 95 % of the recombination occurs in 48 gene-rich regions encompassing 29 % of the physical size of the wheat genome [29]. Therefore, the large genetic distance of distal bins is largely due to the fact that gene-rich regions are often localized in the distal bins with high recombination. One disadvantage of the wheat deletion bin mapping is that loci within chromosome bins cannot ordered [23]. Since the gene based markers were designed from the sequences in the *Ae. tauschii* genetic map, marker order within the bin can be inferred based on their position in the genetic map (Fig. 4).

## Discussion

The use of genomic sequence from related and/or progenitor species, such as *Ae. tauschii*, can facilitate marker development in hexaploid wheat [30]. Comparative genomics studies using sequenced genomes to infer marker and trait location in an un-sequenced genome have been successful to a limited degree. The use of rice, sorghum, maize, and *Brachypodium* genome sequences have been extremely useful for synteny based applications and identification of shared genes and traits [31]. However, non-coding regions are not well conserved even between closely related species. Conservation is so limited that a closely related species such as *Ae. speltoides* does not conserve the majority of repetitive elements as found in *Ae. tauschii* [32]. Evolution of the individual genomes of hexaploid wheat has resulted in similarity in gene content and order, but unique in transposable element content and arrangement. This distinct arrangement of the repetitive elements in the individual genomes can be leveraged to develop genome specific repeat junction markers. These markers are numerous and could potentially be used in platforms designed for high-density marker evaluation [10–12].

Array based markers provide a number of benefits over single marker systems [33]. In this study, we developed the NimbleGen array containing both gene-based and repeat junction-based probes for mapping of the hexaploid wheat genome. Results indicated that 82 % of the NimbleGen mapping data agrees with the sequencing results with respect to chromosome assignment. When mapping probes to specific deletion bins, 60 % of NimbleGen data agreed with the previous wheat EST deletion bin map. Although the discrepancy was not investigated in this study, recently, Belova et al. [20] identified twelve markers showing different bin locations with the two bin mapping methods, and using this data, re-analyzed the bin locations with specific PCR primers. Their result indicated that the PCR method supported the NimbleGen data in eleven markers, suggesting its high level of accuracy for bin assignment [20].

In this study, hybridization of the NimbleGen array with nulli-tetrasomic and deletion bin lines assigned 41,610 probes (repeat junction sites + genes) to individual chromosomes and 16,063 probes to individual deletion bins. The difference in number of probes assigning between nulli-tetrasomic and deletion bin assignment is due partially to the incomplete coverage of the chromosomes by deletion bins. Deletion bins would have provided complete coverage if we had used the ditelosomic stocks to assign markers to individual chromosome arms. The deletion lines used in this study had coverage of ~50 % to 60 % (~2.5Gb) of the D genome and these lines represented mostly the gene rich regions of the chromosomes, where we found almost similar distribution of genes as well as repeat junction sites. The incomplete coverage may largely account for approximately 24,000 probes that were assigned to nulli-tetrasomic lines, but missed by the deletion bins. However, some of the probes may be undetected due to the variation from the labeling method to a technical error during processing resulting in a scratched slide and therefore a loss of statistical significance. In typical comparative genomic hybridization systems, multiple probes are used per gene or locus. In this case, loss of some markers due to labeling variation can be disregarded since there are multiple probes that can be used to evaluate the presence or absence of the gene or locus. However, for repeat junction sequences, we had only a single probe representing each repeat junction and therefore have higher loss of probe assignments between experiments. This could partially explain why a higher number of repeat junction sequence probes failed to map to deletion bins. However, once the probes have been placed into deletion bins and oriented spatially then physically linked markers can be analyzed together in order to identify presence/absence variations.

The development of a high-resolution integrated physical and genetic map for the hexaploid wheat genome is crucial for generating a high-quality wheat genome assembly. Coverage of the genome for construction of a deletion bin map can be conservatively estimated by the number of probes mapping to individual deletion bins. In this study, we mapped 16,063 probes (10,892 gene based probes + 5,171 repeat junction sites) to the delineated deletion bins. This would produce a ratio of ~3.2 markers/Mb based on the 5 Gb *Ae. tauschii* genome. This ratio should increase to ~6.4 markers/Mb in the covered region since the deletion bins used for mapping represented 2.5 Gb in size. This resolution could provide sufficient marker density to aid in various genetic and genomics studies in wheat. For example, RH mapping which relies on physical breakage of chromosomes and is independent of recombination is a strategy for generate genome maps with more uniform resolution than genetic maps[24, 34, 35]. Therefore, such maps are not only useful for determining the physical distance between two markers, but also essential for studying low recombination regions that can't be easily accessed using genetic recombination methods. Genotyping RH populations are based on the presence or absence of markers. Hence, repeat junction markers will be useful, particularity in polyploidy genomes since they are often genome-specific without the need for further experimental assignment [9]. This type of markers have been used in generating the high resolution RH map for the wheat chromosome 3B [35, 36]. A high-resolution RH map with both gene-based and repeat junction markers can better facilitate anchoring and ordering of the BAC contigs in low recombination and low gene density regions. BAC contigs in these regions might be difficult to anchor onto a genetic map due to the low recombination event between two markers and much fewer genes associated with those BAC contigs. Because of the ubiquitous of transposable elements, BAC contigs likely contain repeat junction sites. If the BAC contigs are identified to contain mapped repeat junction sites, they can be immediately assigned to specific deletion bins. An ongoing project to generate a high resolution RH map using the NimbleGen array will provide additional framework for anchoring and ordering the BAC contigs to accomplish construction of sequence ready physical maps of the D genome of hexaploid wheat [35]. The genome sequence of *Ae. tauschii*, one of the three progenitors of bread wheat, is a useful resource for studying abiotic and biotic stresses and other important traits for wheat improvement [37]. In this study, we mapped the gene markers developed from *Ae. tauschii* physical mapping project [21] onto the deletion bins, hence these markers can be directly linked to physical BAC contigs and their sequences (http://aegilops.wheat.ucdavis.edu/ATGSP/). In addition, since the probe sequences on the NimbleGen array have been validated as common between the D genome of *Ae. tauschii* and Chinese Spring, the resource will be very useful for localizing a trait of interest and its eventual map based cloning study for the D genome of hexaploid wheat.

## Conclusions

In summary, we developed a NimbleGen CGH array and mapped 29,193 repeat junction sites and 12,417 gene based markers to specific D chromosomes of the wheat genomes. Among them, 5,171 RJMs and 10,892 gene probes representing 5,070 genes were mapped to the deletion bins of the D genome. Therefore, in addition to these repeat junction sites, we also mapped more than 2000 genes to the deletion bins as compared to the previous EST deletion bin map (2,962 gene sequences). In bread wheat, aneuploid stocks have been extensively used to assign markers linked with genes/traits to chromosomes, chromosome arms, and their specific bins. One disadvantage of the previous assignment of wheat ESTs to chromosome bins is that loci within each bin cannot be ordered. By utilizing the *Ae. tauschii* genetic map, in this study, the mapped genes within the deletion bins could be easily ordered. The ordered genes as well as repeat junction sites in deletion bin lines can provide a valuable resource for targeted mapping and map based cloning studies of the genes located on the D genome chromosomes of hexaploid wheat. A specific database (http://probes.pw.usda.gov/ATRJM/) was developed to allow users to search for marker/probe sequences within specific deletion bins or individual chromosomes by a simple BLAST analysis or marker ID input. In addition, we showed that 17,453 probes on the NimbleGen array can be linked to sequence contigs in the bread wheat sequence data, with an accumulated sequence length of ~155 Mb, representing ~3.2 % of the D genome. If a probe sequence can be anchored to the Chinese Spring shotgun sequence assembly data, the website will provide a link connecting to the sequence scaffold annotated in the EnsemblPlants database (http://plants.ensembl.org/index.html). This provides additional sequence information surrounding the marker/probe for further analysis. In addition, PCR primers designed to amplify the marker regions are provided and available for download, providing an easy and effective marker system for mapping of individual genes/traits of interest.

## Methods

### Identification of RJMs for NimbleGen array

A 1X Roche 454 shotgun genome sequence of *Ae. tauschii* accession AL8/78 [21] was used to screen and identify repeat junction region sequences using a software pipeline, RJPrimer [8] developed previously in the lab. After the identification of Roche 454 reads containing repeat junction regions, we employed multiple steps in the selection process of repeat junction markers for the NimbleGen array; including trimming of the marker sequences to 52 bp with the repeat junction at the midpoint, removing high-copy junction markers via BLASTN, eliminating sequence errors through sequence validation with Illumina reads, and selection of markers with the GC content ranging 50 % to 65 % and melting temperature ranging from 76 °C to 83 °C.

### Design of high-throughput NimbleGen mapping array

A 135,000 (3X 45,000) probe array was designed using: 31,205 *Ae. tauschii* repeat junction markers and 15,016 gene markers for 6,348 genes. The genes were represented by 1–6 marker probes. Each marker was replicated three times as 52 bp long probes randomly printed on the array and all probes were of a length of 52 bp. Each repeat junction probe was duplicated on the nullisomic-tetrasomic and deletion lines of D genome chromosomes. NimbleGen loading and handling controls were included to standardize the arrays and to identify poor labeling or poor hybridization.

### Plant materials

Seven nullisomic-tetrasomic lines of Chinese Spring, each deficient for one of the seven D genome chromosomes (N1D-T1A, N2D-T2A, N3D-T3A, N4D-T4B, N5D-T5A, N6D-T6A, N7D-T7A) and 40 D chromosome specific deletion bin lines (1DS-1, 1DS-5, 1DL-4, 1DL-2, 2DS-1, 2DS-5, 2DL-3, 2DL-9, 2DL-6, 3DS-3, 3DS-7, 3DS-6, 3DL-2, 3DL-3, 4DS-1, 4DS-3, 4DS-2, 4DL-13, 4DL-12, 4DL-9, 5DS-1, 5DS-5, 5DS-2, 5DL-7, 5DL-1, 5DL-9, 5DL-5, 6DS-2, 6DS-4, 6DS-6, 6DL-6, 6DL-1, 6DL-12, 6DL-11, 7DS-5, 7DS-4, 7DS-6, 7DL-5, 7DL-2, 7DL-3) were used in this study. DNA from the leaf tissues from the nullisomic-tetrasomic as well as deletion bin lines was isolated and purified using previously described methods [38–40]. Deletion bin lines used in this study represent ~50 % of the D genome and were informative mostly for telomeric regions of the D chromosomes.

### NimbleGene Array hybridization

The NimbleGen array was hybridized in duplicate with Cy3 labeled seven nullisomic-tetrasomic lines, deletion bin lines for D genome chromosomes, and control reference Chinese Spring; as well as Cy5 labeled reference line Chinese Spring. All hybridizations were carried out at 42 °C. All buffers and wash conditions were performed using NimbleGen methods and protocols (www.nimblegen.com).

### NimbeGen array data analysis

Array image files were analyzed using Roche DEVA software and signal intensities were quartile normalized using the program Expander (http://acgt.cs.tau.ac.il/expander/) [41]. The individual normalized Cy3 signals were averaged between the three replicates and the average signal was compared to the averaged reference Chinese Spring Cy5 signals. The *P*-value for each probe was calculated using a Student’s *T*-test. Each probe was assigned to a deleted chromosome using the following criteria: a minimum decrease of signal of 20 % compared with the reference signal and a P-value <0.05. A decrease of at least 20 % was determined by comparing map positions assigned by the NimbleGen array with map positions determined by PCR (data not shown) or known gene markers; this analysis suggested a cutoff signal of at least 20 % for accurate results.

### Availability of supporting data

The data sets supporting the results of this article are included within the article (and its additional files). Additionally, a public database was developed for this project to allow users to search for marker sequences and their map locations at the website http://probes.pw.usda.gov/ATRJM/. The NimbleGen hybridization data is available in the NCBI's Gene Expression Omnibus (GEO) database repository (http://www.ncbi.nlm.nih.gov/geo/) with the accession number GSE71190.

## Additional file

Additional file 1: Supplement T1. Comparison of NimbleGen mapping data with the previous wheat EST deletion bin map. (XLSX 20 kb)

## Abbreviations

BAC: Bacterial artificial chromosome
CGH: Comparative genomic hybridizations
CNV: Copy number variants
CS: Chinese Spring
EST: Expressed sequence tag
ISBP: Insertion site-based polymorphism
PAV: Presence/absence variations
QTL: Quantitative trait loci
RJMs: Repeat junction-based markers
SNP: Single nucleotide polymorphism
